# Supplementary material for: Climate warming reduces the temporal stability of plant community biomass production
Source: Nat Commun. 2017 May 10;8:15378. doi: 10.1038/ncomms15378 (PMC5436222; doi:10.1038/ncomms15378)
Supplement: Supplementary Information — Supplementary Figures and Supplementary Tables [file ncomms15378-s1.pdf]

Supplementary Table 1. Results (*F*-value) of linear mixed-effects models (LMMs) testing the effect of warming (W), precipitation (P), year (Y) and their interactions on community biomass, species richness, Simpson's dominance, and the result of LMMs testing the effects of warming (W), precipitation (P) and their interactions on community biomass stability, species asynchrony, dominant species stability, common species stability and rare species stability (n=5).

	Biomass (g m^{-2})	Richness	Dominance	Community biomass stability	Species asynchrony	Dominant species stability	Common Species stability	Rare species Stability
W	0.04	**46.66*** **	**8.07** **	**4.86***	**7.74***	1.54	1.88	0.15
P	**32.12*** **	**31.20*** **	**4.43***	1.93	1.03	1.41	0.12	0.79
Y	**22.29*** **	**29.64*** **	**5.72** **	—	—	—	—	—
W*P	**4.05***	2.95	2.21	0.10	0.18	0.05	0.96	1.06
W*Y	1.26	**3.76***	2.55	—	—	—	—	—
P*Y	1.18	**2.22***	**2.33***	—	—	—	—	—
W*P*Y	1.18	1.60	1.25	—	—	—	—	—

**P*<0.05, ***P*<0.01, ****P*<0.001. Significant results (*P*<0.05) are bolded.

Supplementary Table 2. Results of linear mixed-effects models testing the effect of warming (W), precipitation (P) and their interactions on the stability of four different functional groups (grasses, sedges, legumes and forbs) (n=5).

	Grasses stability		Sedges stability		Legumes stability		Forbs stability	
	F-value	*P*	*F-value*	*P*	*F-value*	*P*	*F-value*	*P*
W	0.64	0.43	1.82	0.19	0.77	0.39	1.27	0.27
P	0.79	0.47	2.48	0.11	0.37	0.70	1.52	0.24
W*P	0.12	0.35	1.03	0.38	0.19	0.83	0.63	0.54

Supplementary Table 3. Results of the structural equation model of warming and precipitation treatment effects on community temporal stability through all plausible pathways. Given are the standardized path coefficients, standard errors of regression (S.E.), Z-values, and the levels of significance for the regression (n=30).

Path			Standard coefficient	S.E.	Z-value	P(>| z |)
Species richness	←	Warming	-0.56	0.60	-5.98	0.00
Species richness	←	Drought	-0.51	0.74	-4.67	0.00
Species richness	←	Wet	0.22	0.74	2.00	0.05
Species dominance	←	Warming	-0.34	0.02	-2.24	0.03
Species dominance	←	Drought	-0.19	0.02	-1.06	0.29
Species dominance	←	Wet	0.29	0.02	1.64	0.10
Dominant species stability	←	Warming	-0.14	1.16	-0.53	0.60
Dominant species stability	←	Drought	-0.02	1.23	-0.06	0.95
Dominant species stability	←	Wet	0.21	1.02	0.96	0.34
Dominant species stability	←	Species richness	0.11	0.23	0.34	0.73
Dominant species stability	←	Species dominance	0.09	8.15	0.45	0.66
Species asynchrony	←	Warming	-0.55	0.05	-2.33	0.02
Species asynchrony	←	Drought	-0.29	0.06	-1.20	0.23
Species asynchrony	←	Wet	0.09	0.05	0.47	0.64
Species asynchrony	←	Species richness	-0.13	0.01	-0.44	0.66
Species asynchrony	←	Species dominance	-0.05	0.38	-0.28	0.78
Community stability	←	Warming	-0.01	0.52	-0.09	0.93
Community stability	←	Drought	-0.17	0.51	-1.19	0.24
Community stability	←	Wet	-0.16	0.43	-1.35	0.18
Community stability	←	Species richness	0.02	0.09	0.12	0.91
Community stability	←	Species dominance	-0.07	3.33	-0.60	0.55
Community stability	←	Species asynchrony	0.49	1.61	4.52	0.00
Community stability	←	Dominant species stability	0.62	0.07	6.24	0.00

Supplementary Table 4. The name and relative abundance (RA) of dominant, common and rare species in our study (53 species in total). Nomenclature follows the editorial committee of Chinese plant records.

Dominant species		Common species		Rare species	
Latin name	RA (%)	Latin name	RA (%)	Latin name	RA (%)
Stipa aliena	43.35	*Kobresia humilis*	4.41	*Gentiana farreri*	0.97
Elymus nutans	5.38	*Poa annua*	4.22	*Viola kunawarensis*	0.93
		Oxytropis kansuensis	4.12	*Festuca rubra*	0.92
		Deyeuxia flavens	3.59	*Lancea tibetica*	0.79
		Tibetia himalaica	3.27	*Leontopodium nanum*	0.77
		Saussurea pulchra	3.05	*Polygonum viviparum*	0.74
		Kobresia capillifolia	2.36	*Parnassia trinervis*	0.63
		Thalictrum aquilegifolium	2.19	*Potentilla bifurca*	0.62
		Euphrasia regelii	2.16	*Aster diplostephioides*	0.56
		Delphinium grandiflorum	1.71	*Gentianopsis paludosa*	0.49
		Medicago archiducis-nicolai	1.60	*Koeleria litvinowii*	0.43
		Carex przewalskii	1.57	*Ranunculus tanguticus*	0.38
		Scirpus distigmaticus	1.56	*Swertia tetraptera*	0.36
		Gentiana aristata	1.51	*Potentilla nivea*	0.36
		Helictotrichon tibeticum	1.41	*Lomatogonium rotatum*	0.33
		Gentiana straminea	1.39	*Anemone obtusiloba*	0.30
				Thermopsis lanceolata	0.28
				Saussurea nigrescens	0.26
				Taraxacum mongolicum	0.21
				Iris lactea	0.21
				Anaphalis lactea	0.16
				Astragalus membranaceus	0.11
				Stellaria media	0.11
				Potentilla anserina	0.10
				Ptilagrostis dichotoma	0.09
				Pedicularis verticillata	0.02
				Lonicera minuta	0.02
				Glaux maritima	0.01
				Veronica ciliata	<0.01
				Gentiana pudica	<0.01
				Notopterygium incisum	<0.01
				Morina kokonorica	<0.01
				Ligularia sagitta	<0.01
				Potentilla fruticosa	<0.01
				Ajania tenuifolia	<0.01

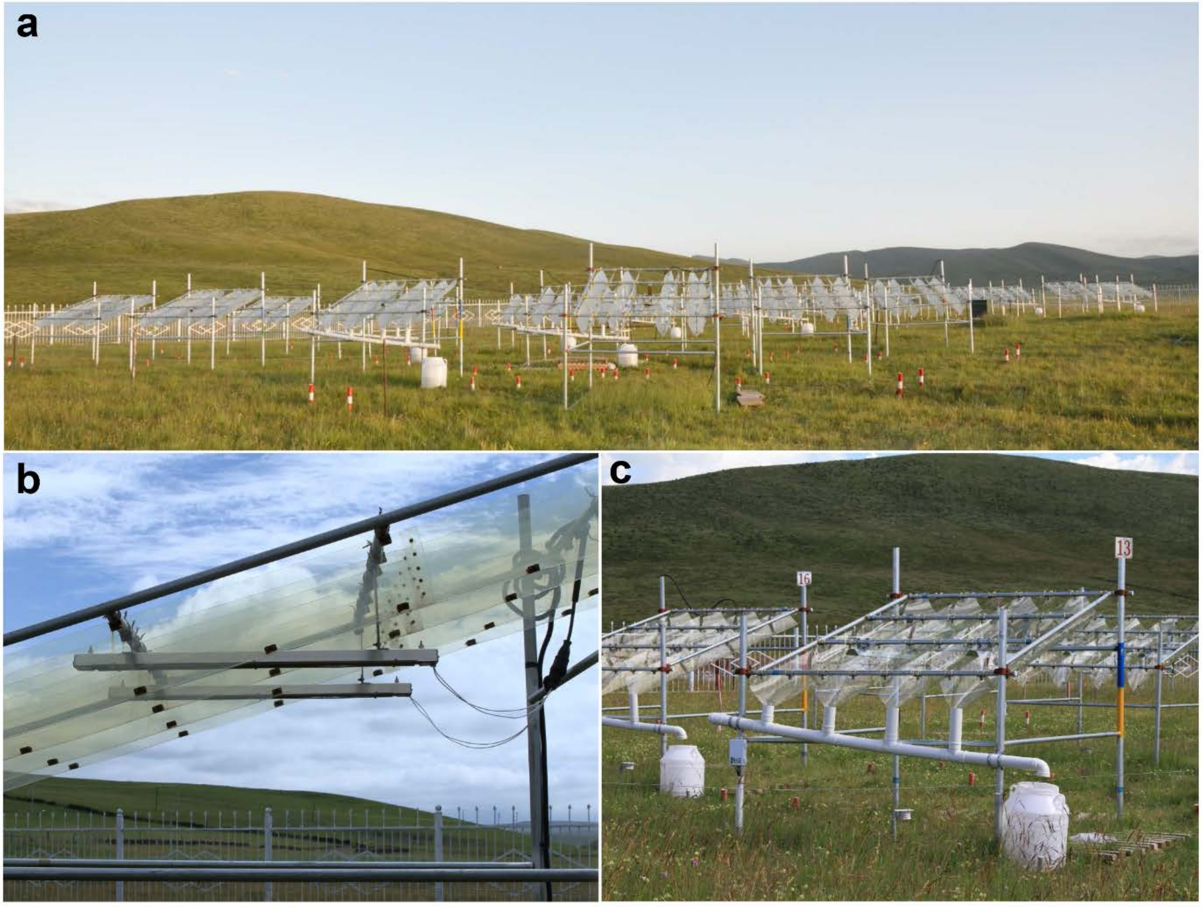

Supplementary Figure 1. Photos of our experimental site The experiment was established in 2011 in an alpine grassland on the Tibetan Plateau, using a randomized block design with warming and altered precipitation as the main treatment factors. Shown are (a) The overall view of the experimental plots; (b) plots experiencing warming; and (c) plots experiencing precipitation alteration. Photo credit: Jin-Sheng He.

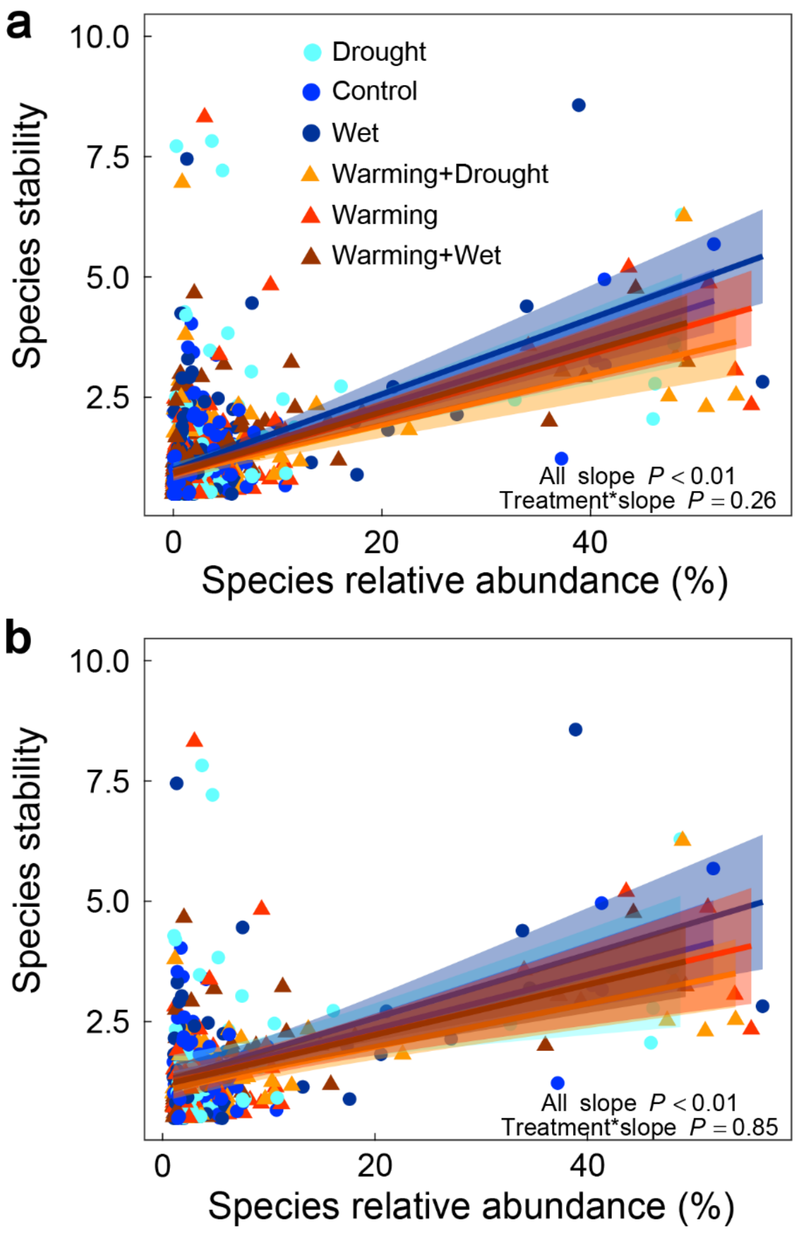

Supplementary Figure 2. Relationships between species stability and relative abundance in different experimental treatments Data shown are for (a) all species (dominant, common and rare species) (linear regressions; n=1,079; all P<0.01); (b) dominant and common species only (linear regressions; n=422; all P<0.01). The solid lines are significant regression lines, shaded areas represent 95% confidence intervals.

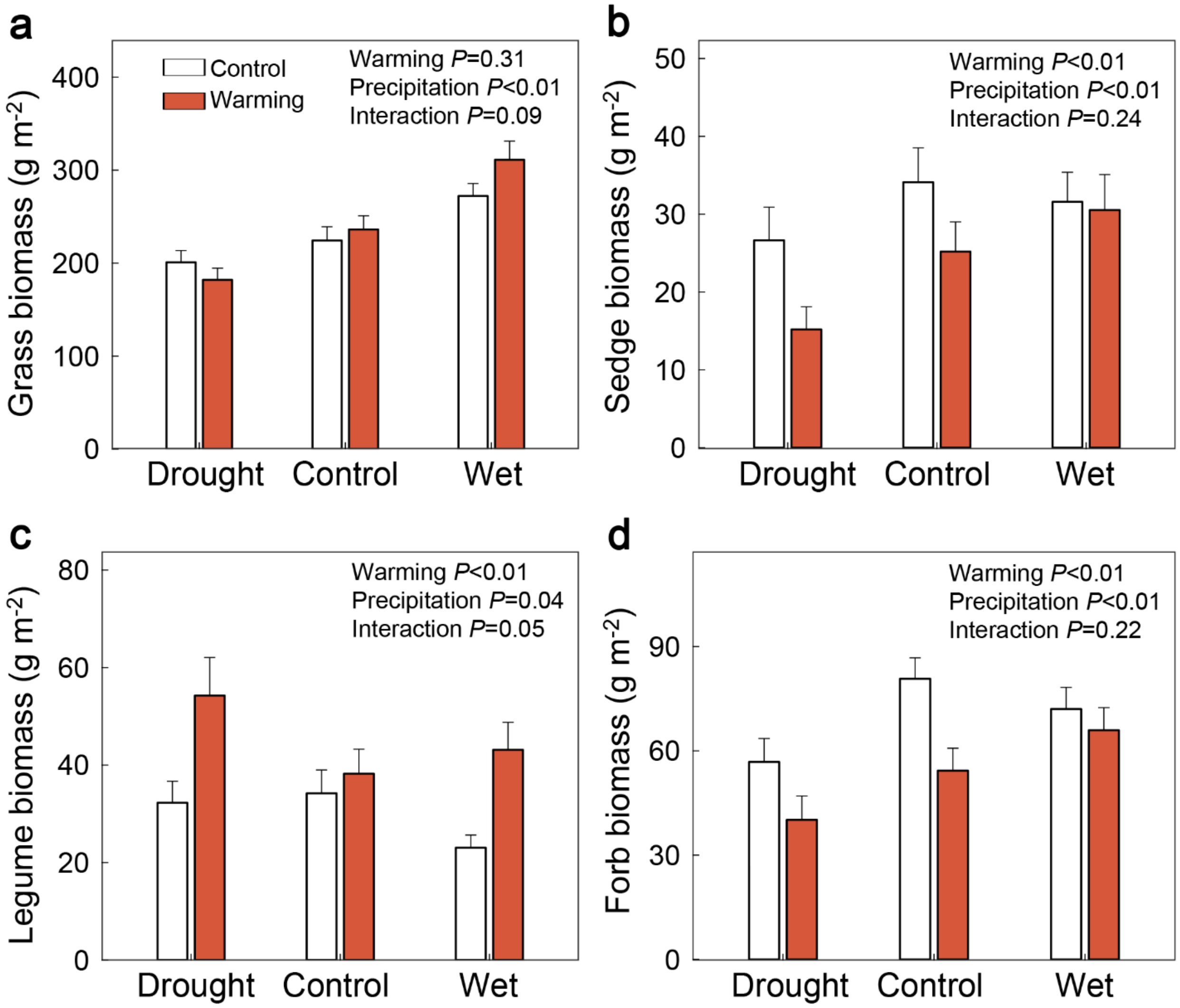

Supplementary Figure 3. Functional group biomass in different experimental treatments

Shown are (a) grass biomass (Linear mixed-effects model; warming: P=0.31; precipitation: P<0.01; interaction: P=0.09); (b) sedge biomass (Linear mixed-effects model; warming: P<0.01; precipitation: P<0.01; interaction: P=0.24); (c) legume biomass (Linear mixed-effects model; warming: P<0.01; precipitation: P=0.04; interaction: P=0.05) and (d) forb biomass (Linear mixed-effects model; warming: P<0.01; precipitation: P<0.01; interaction: P=0.22). Drought, 50% reduction in precipitation compared to control; Wet, 50% increase in precipitation compared to control. Vertical bars represent the s.e.m (n=5).

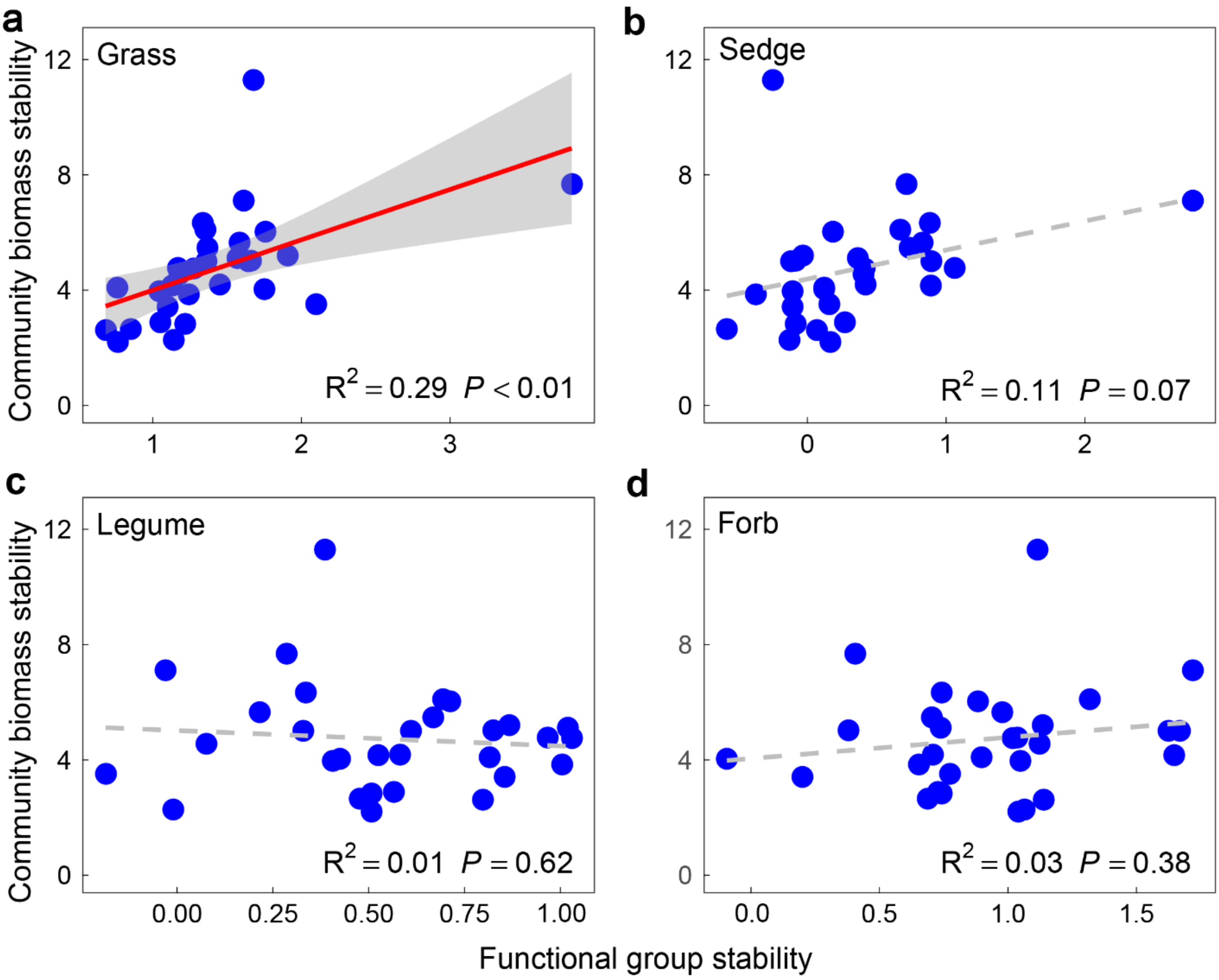

Supplementary Figure 4. Relationships between functional groups stability and temporal stability of community biomass Shown are (a) grass stability (linear regression; P<0.01); (b) sedge stability (linear regression; P=0.07); (c) legume stability (linear regression; P=0.62); and (d) forb stability (linear regression; P=0.38). Values of functional groups stability were log-transformed. The red solid lines are significant regression lines, and the gray dashed lines are non-significant regression lines. Each blue circles represents an experimental plot (n=30), shaded areas represent 95% confidence intervals.

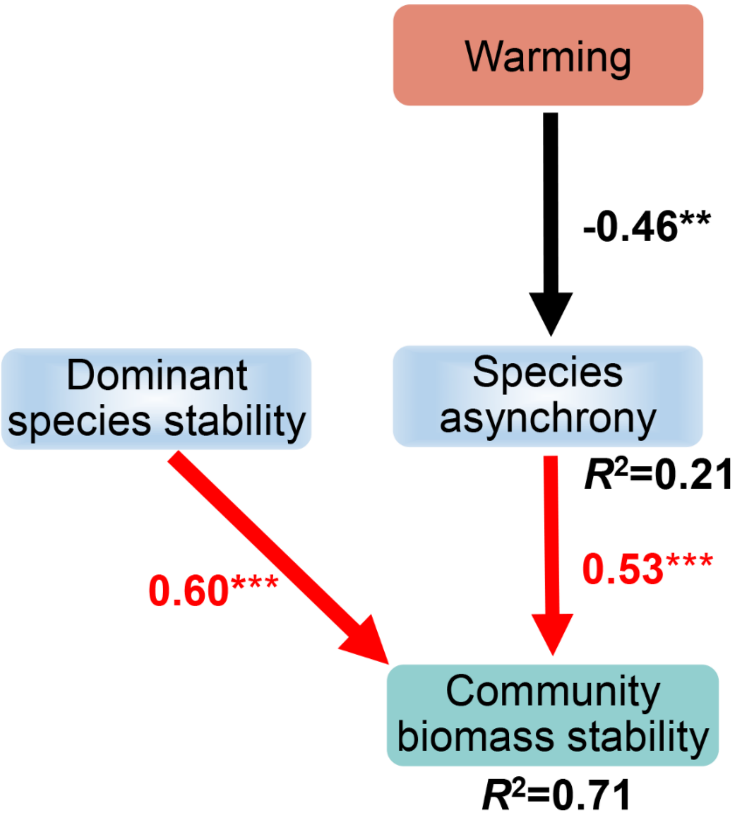

Supplementary Figure 5. The final structural equation model relating experimental treatments to biomass temporal stability The model indicates that warming reduces community biomass temporal stability by reducing species asynchrony. Red and black arrows represent significant positive and negative pathways, respectively. Numbers indicate the standard path coefficients. Arrow width is proportional to the strength of the relationship. R^2 represent the proportion of variance explained for each dependent variable in the model. *P<0.05, **P<0.01, ***P<0.001. χ^2=5.726, P=0.057; RMSEA=0.25, P=0.07; AIC=210.98.

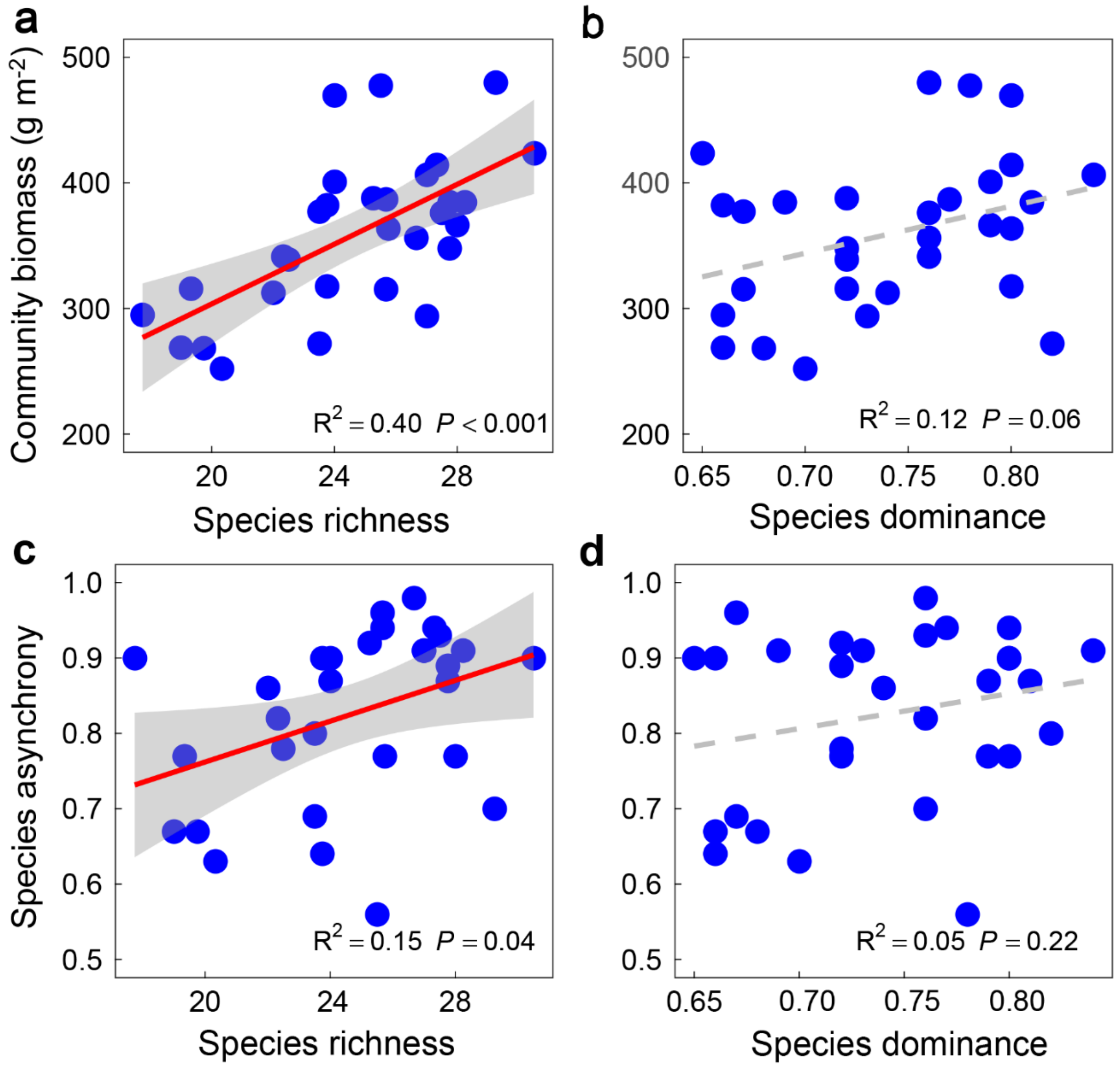

Supplementary Figure 6. Relationships between species richness/community dominance and community biomass/species asynchrony Shown are species richness in relation to (a) biomass (linear regression; *P*<0.001) and (c) species asynchrony (linear regression; *P*=0.04); community dominance in relation to (b) community biomass (linear regression; *P*=0.06) and (d) species asynchrony (linear regression; *P*=0.22). The red solid lines are significant regression lines, and the gray dashed lines are non-significant regression lines. Each blue circle represents an experimental plot (n=30), shaded areas represent the 95% confidence interval.

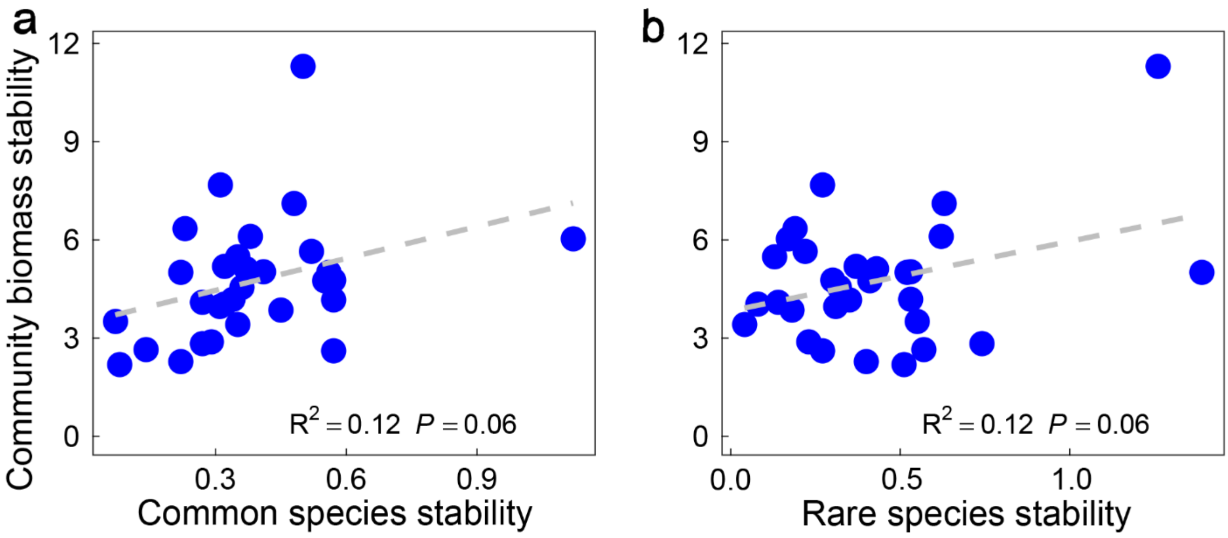

Supplementary Figure 7. Relationships between subordinate species stability and temporal stability of community biomass Shown are (a) common species stability and (b) rare species stability. Neither relationship was significant (linear regression; *P*=0.06 for both). Common and rare species stability were log-transformed. The gray dashed lines are non-significant regression lines. Each blue circle represents an experimental plot (n=30).
